# Optimizing Communication of Radiation Exposure in Medical Imaging, the Radiologist Challenge

**DOI:** 10.3390/tomography9020057

**Published:** 2023-03-23

**Authors:** Chiara Pozzessere

**Affiliations:** 1 Department of Diagnostic and Interventional Radiology, Lausanne University Hospital (CHUV), 1011 Lausanne, Switzerland; chiara.pozzessere@chuv.ch; 2 Faculty of Biology and Medicine, University of Lausanne (UNIL), 1011 Lausanne, Switzerland

**Keywords:** radiation protection, radiation exposure, computed tomography, patient communication, patient information, ChatGPT

## 1. Introduction

Since I started my residency program in Radiology, I have been committed to promoting radiation protection, paying particular attention to the justification and optimization of the examinations. With great confidence, I can argue the risks and benefits of the requested examination with the referring physicians. Nevertheless, I must admit to being still a little uncomfortable when I have to inform the patients and help them to make the right decision in specific cases. This is especially true when it comes to pregnant women, women of childbearing age or children. One must be prepared to confidently answer questions such as this: “What happens to my baby if I undergo this examination?”. Once, a woman in her 4th month of pregnancy who had had a head injury, despite headache and visual disturbances, did not want to undergo a head Computed Tomography (CT) scan, saying: “My baby’s life is more important than mine”. It was hard to persuade her of the importance of that scan while reassuring her about the potential, non-negligible but low, risks to the foetus. However, an episode that happened a few years ago particularly impressed me. An approximately 8-year-old child had fallen from a tree from a height of about 2 m; after a highly suspicious ultrasound examination, the emergency department (ED) physician requested an abdominal CT scan to rule out traumatic injuries to the abdominal organs and vertebral fractures as well as a chest radiography. I introduced myself to both the mother and the child to collect their informed consent. While briefly explaining the goal of the examination, the mother started asking me specific questions about the damage of ionizing radiation for their child. I wanted to provide accurate information, and I explained that the dose delivered would have been carefully reduced as much as possible and that the benefits of the scan outweighed the risks. However, she chased me with hundreds of specific questions on the effects of the radiation: “I know that radiations can cause DNA mutations, what can happen to my child?”, and I naively replied, “Hypothetically within 10–20 years a tumour can arise but these data are speculative, there are no scientific data for this radiation exposure”. At that sentence, the child burst into tears and started screaming, he no longer wanted to undergo the examination. I had fallen into the trap: the child’s father had recently died of cancer. I remember how clumsy I must have sounded when trying to reassure both the mother and the child. Although I could finally calm the child, obtain the consent and perform the scan, that episode impressed me and made me think about the challenges of communicating radiation risks and benefits of radiological examinations.

## 2. The Challenge

The increased use of CT, responsible for up to 67% of radiation exposure in medical imaging, represents a major concern [1]. As highlighted by a large multicentre study, a cumulative effective dose above 100 mSv is delivered to more than 1% of patients undergoing multiple CT examinations [2].

Therefore, radiologists and radiographers are at the forefront of the commitment to radiation protection. Radiologists and technologists are trained in radiation protection and receive continuing education throughout their careers. In particular, radiologists are called to ensure the justification and optimization of the examinations and to inform patients about the benefits and risks associated with imaging, as established by the Council Directive 2013/59/EURATOM. In particular, under chapter 7, article 57, paragraph (d), it is stated that *“wherever practicable and prior to the exposure taking place, the practitioner or the referrer, as specified by Member States, ensures that the patient or their representative is provided with adequate information relating to the benefits and risks associated with the radiation dose from the medical exposure.”* [3].

However, as said, patient communication is not easy to put into practice. Although radiologists should have all the skills and expertise, explaining a stochastic but disconcerting risk to unaware subjects is tricky. Patients want to be informed about radiation-related risks, but they have a poor understanding of radiation exposure and the risk of medical imaging [4]. The information to address is as follows: (i) there is a risk of cancer, which is a potentially lethal disease; (ii) the risk is supposed to be very low, maybe negligible below 100 mSv; and (iii) the risk is remote, delayed by 10–20 years. The uncertainty of this frightening information and the absence of data available in support makes the patient information and communication difficult [5]. Consequently, the absence of keys for correct communication may lead to: (1) a lack of communication of the risks to avoid useless discussion and concern; (2) inappropriately understating the risks to reassure the patient; and (3) overloading the patient with an excessively scientific lexicon and unnecessary details, miscommunicating the essential information. Let us suppose another clinical scenario: the surgeon who illustrates short- and long-term complications of a surgical intervention. Everything is concrete and real to the patient. First, the intervention itself is scaring compared to an apparently safe “picture”. Secondly, the surgeon presents real data—percentage and timing—of the complications compared to an uncertain radiological risk.

## 3. Strategies for Optimizing Communication

So, how to overcome this issue? First, should the information be conveyed to the patient in a written or oral form? Written informed consent could address all the information in a pre-formed, easy-to-read document, and it has a certain legal value, but, unfortunately, its signature does not reflect real patient understanding. Therefore, oral communication with the patient is important to provide information tailored to the educational level of each patient, allowing for the immediate clarification of doubts and also the answering of questions.

The keys to providing correct information and communication are familiarity with the topic, cordiality, transparency and understandability of the concept. In clinical situations, patients are often stressed about their health condition, and their attitude may reflect this. They may overestimate or minimize negative information, or they may not listen to the entire speech, focusing on what they want to hear. That is why cordiality, transparency and understandability can increase trust in the stakeholder’s speech. Technical and medical words should be explained through simple terms, avoiding acronyms and technical jargon [6]. Although some limitations arise from individual perception, it would be useful and impactful comparing the amount of radiation released by the examination and/or the related risk with something with which the patient is familiar [7]. Transcontinental flight, individual average annual background radiation, cigarette smoking or driving accident-related risk and lifetime cancer risk can be used as examples by tailoring these to the patient’s characteristics, such as their age, educational level and attitude through the use of a risk-ranking scale [7,8,9]. Providing two or more examples may increase their confidence, as will separately reporting all the benefits of the examination to exhaustively describe the benefit–risk balance [7]. Patients want to be reassured of the fact that we are taking care of them and that we are scrupulously committed to our work. Another communication tip is to show the machine that will be used, explaining that scanner technology itself limits the radiation exposition and finally ensuring that the radiologist and the radiographer would further optimize the parameters to obtain diagnostic imaging at the lowest dose possible for the patient’s specific clinical question. In case dose management systems (DMSs) are implemented in the facility, specifying that DMSs monitor the radiation dose delivered on a regular basis to check for compliance with the diagnostic reference levels (DRLs) would strengthen the radiology department’s commitment to radiation protection [10].

It is now easy to understand that, in the example reported above, I made several mistakes. Although I introduced myself and I was calm and exhaustive, I was vague and theoretical, and I showed difficulties in conveying technical and medical concepts in a friendly way. Moreover, I did not lead the conversation, allowing the patient’s mother to direct the conversation. I should have introduced the benefits of the examination and then briefly explained that the radiation exposure would have been very low by providing a couple of reassuring examples for comparison. Importantly, in this case, I did not highlight my personal commitment to significantly reducing the dose by optimizing the scanner’s parameters.

The ongoing evolving improvement of artificial intelligence (AI) makes it conceivable to use it in this setting. In recent years, generative pre-trained transformer (GPT)-based models, AI tools generating large language models (LLMs), have been extensively testing and developing in healthcare at several levels, such as research, education, clinical decisions and patient communication [11,12,13]. Already ChatGPT, variant of the third GPT generation, has been trained to understand and generate language for conversation with users [12,13,14]. Now GPT-4, the newly released version, promises to create more sophisticated and capable language models. Although these AI models have several limitations, it may be interesting to improve GPT in radiation protection, including healthcare professional education and patient information and to assist radiologists and radiographers in effectively communicating the risk–benefit balance of radiation exposure in radiological examination.

## 4. Conclusions

In conclusion, communicating the radiation risk related to medical imaging is a legal duty and a challenge that radiologist face in daily clinical activity. Its compliance is an engagement to improve the quality of care, patient information and active participation. However, explaining the uncertain likelihood of the risk of a non-painful simple “picture” in easy, non-technical words is often difficult, leading to a lack of information or miscommunication. Providing comprehensive and comforting explanations with a positive attitude by avoiding technical and negative terms, using analogies and examples according to patients’ age, education and attitude while describing the benefits and risks of the examination and showing a personal commitment to optimizing the acquisition parameters are tips to improving radiation exposure communication. In the future, it would be expected that AI tools, such as GPT, would be improved, not to substitute healthcare professionalism, but to serve as a support at several levels, including radiation protection education and information as well as facilitating effective and exhaustive communication of radiation exposure in radiological examination.

## Abbreviations

AI	Artificial intelligence
CT	Computed Tomography
DLRs	Diagnostic reference levels
DMS	Dose management systems
DNA	Deoxyribonucleic Acid
ED	Emergency Department
GPT	Generative pre-trained transformer
LLM	Large language model
